# A Computational Framework to Predict Calvarial Growth: Optimising Management of Sagittal Craniosynostosis

**DOI:** 10.3389/fbioe.2022.913190

**Published:** 2022-05-24

**Authors:** Connor Cross, Roman H. Khonsari, Giovanna Patermoster, Eric Arnaud, Dawid Larysz, Lars Kölby, David Johnson, Yiannis Ventikos, Mehran Moazen

**Affiliations:** ^1^ Department of Mechanical Engineering, University College London, London, United Kingdom; ^2^ Department of Maxillofacial Surgery and Plastic Surgery, Necker—Enfants Malades Hospital, Assistance Publique—Hôpitaux de Paris, Paris, France; ^3^ Department of Neurosurgery, Craniofacial Surgery Unit, Necker—Enfants Malades Hospital, Assistance Publique—Hôpitaux de Paris, Paris, France; ^4^ Department of Head and Neck Surgery for Children and Adolescents, University of Warmia and Mazury in Olsztyn, Prof. St. Popowski Regional Specialized Children’s Hospital, Olsztyn, Poland; ^5^ Department of Plastic Surgery, Sahlgrenska University Hospital, University of Gothenburg, Gothenburg, Sweden; ^6^ Oxford Craniofacial Unit, Oxford University Hospital, Oxford, United Kingdom

**Keywords:** calvarial bones, sutures, skull growth, sagittal synostosis, finite element method, biomechanics

## Abstract

The neonate skull consists of several bony plates, connected by fibrous soft tissue called sutures. Premature fusion of sutures is a medical condition known as craniosynostosis. Sagittal synostosis, caused by premature fusion of the sagittal suture, is the most common form of this condition. The optimum management of this condition is an ongoing debate in the craniofacial community while aspects of the biomechanics and mechanobiology are not well understood. Here, we describe a computational framework that enables us to predict and compare the calvarial growth following different reconstruction techniques for the management of sagittal synostosis. Our results demonstrate how different reconstruction techniques interact with the increasing intracranial volume. The framework proposed here can be used to inform optimum management of different forms of craniosynostosis, minimising the risk of functional consequences and secondary surgery.

## Introduction

The neonate skull consists of several bony plates, connected by fibrous soft tissues along their edges, called sutures (Opperman, 2000; Herring, 2008; Richtsmeier and Flaherty, 2013). Sutures facilitate birth and accommodate rapid brain growth in the first year of life (Herring, 2008; Richtsmeier and Flaherty, 2013). Premature fusion of sutures is a medical condition known as craniosynostosis (Herring, 2008; Richtsmeier and Flaherty, 2013; Johnson and Wilkie, 2011). The most common form of this condition is sagittal synostosis (SS) caused by premature fusion of the sagittal suture, occurring in approximately 3 in every 10,000 births. This condition leads to bi-temporal narrowing and excessive anteroposterior growth of the skull with frontal and occipital bossing (Johnson and Wilkie, 2011; Mathijssen, 2015).

The management of SS involves surgical remodelling of the calvaria. The underlying aims of the surgery are to normalise the head shape and relieve the constraint on the growing brain, thus decreasing the potentially elevated intracranial pressure (ICP) (Mathijssen, 2015). Several techniques have been developed and used over the years across the world for the management of SS (Mathijssen, 2015; Jane et al., 1978; Jimenez & Barone, 2012; Simpson et al., 2017). These range from less invasive methods such as strip craniotomy and spring-mediated cranioplasty which are usually performed before 6 months of age, to more invasive approaches such as total vault remodelling which are usually performed at the age of about 12 months (Taylor and Maugans, 2011; van Veelen et al., 2016; Fischer et al., 2016; Gailey et al., 2021).

There is a growing number of clinical studies comparing the outcomes of different techniques for SS (Taylor and Maugans, 2011; van Veelen et al., 2016; Fischer et al., 2016; Gailey et al., 2021). Nonetheless, our fundamental understanding of how different reconstruction methods interact with the growing brain is still limited. This is crucial as one of the key factors during early craniofacial development is the load arising from a growing brain. If this load is not accommodated by the reconstructed skull, it can constrain brain growth, leading to raised ICP and a possible risk of re-operation (Thomas et al., 2015). Computational models are a promising tool to predict calvarial growth and optimise the management of craniosynostosis (Weickenmeier et al., 2017; Lee et al., 2017; Malde et al., 2019).

In a series of studies, we have previously developed a validated computational model based on the finite element method that enables us to predict the radial expansion of the calvaria as well as the bone formation at the cranial sutures in mice and humans (Marghoub et al., 2018; Marghoub et al., 2019; Libby et al., 2017; Malde et al., 2020; Cross et al., 2021a,b). In this current work, 1) we present a computational framework that can be used to advance the treatment of various forms of craniosynostosis; 2) we inform and optimise the clinical management of sagittal craniosynostosis. We first virtually reconstructed the calvaria of a patient at 4 months of age, using 9 different techniques. Then we predicted the calvarial growth up to 76 months of age across all treatment options. Finally, we compared the overall morphology of the calvaria, level of contact pressure across the intracranial volume (ICV), and the pattern of bone formation between the considered techniques. To the best of our knowledge, the computational framework presented here is the first of its kind to predict the calvarial growth and the first steps toward the biomechanical optimisation of the clinical management of craniosynostosis. Both these elements are novel and constitute the main contributions of this study.

## Materials and Methods

### Model Development

**FIGURE 1:**
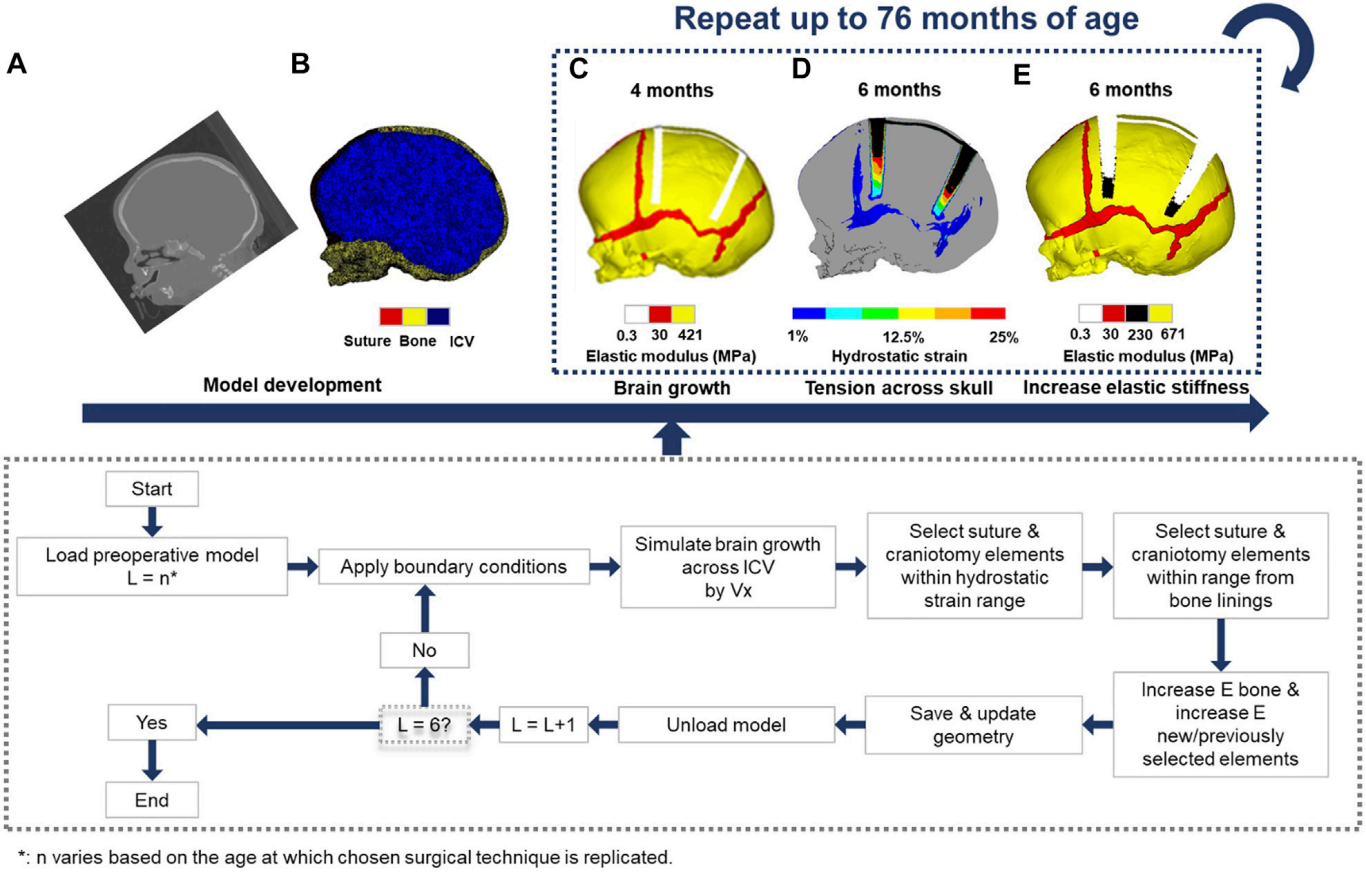
Using CT -scan data at an initial preoperative age of 4 months **(A)**, a 3D finite element model was developed **(B)**. The chosen surgical technique at the appropriate age was performed **(C)**. The intracranial volume was then expanded to a specified volume in 6 load steps **(D)**. Elements across the sutures and craniotomies were then selected based on the level of hydrostatic strain and/or radii process from the bone lining, following the algorithm described in the flow chart. This process was repeated while updating the material properties and geometry of the model at each load step until the final load step has been reached **(E)**. The intracranial volume at the final load step was equivalent to 76 months of age.

The overall computational methodology implemented here is illustrated in Figure 1. Computed tomography (CT) images of a single preoperative sagittal craniosynostosis child skull at the age of 4 months were obtained from the Hôpital Necker–Enfants Malades Craniofacial Surgery Unit (Centre de Référence Maladies Rares Craniosténoses et Malformations Craniofaciales CRANIOST, Paris, France) for 3D model development. Full ethical protocol was approved by the institutional review board, committee, and the patients’ guardians. The images had a voxel size of 0.625 mm across all axes.

The image processing software, Avizo (V9.2.0; Thermo Fisher Scientific, Mass, United States) was used for manual and automatic compartmentalisation of highlighted internal and external tissues. Here, the calvarial bones, sutures, and the complete intracranial volume (ICV) were segmented. The specifics of craniotomies performed across the model were based on the common clinical practices of the authors of this study. The model was then used to create a 3D mesh consisting of approximately four million quadratic tetrahedral elements suitable for finite element analysis using the ANSYS platform (ANSYS V19.0; Canonsburg, PA, United States).

### Boundary and Interface Conditions

Isotropic material properties were assigned to all segmented components. The calvarial bones and sutures were assumed to have a linear elastic modulus of 421 MPa and 30 MPa, respectively (Coats and Margulies, 2006; Moazen et al., 2015), whereas the ICV and craniotomies elastic modulus was defined as 10 and 0.3 MPa, values adopted from our previous sensitivity study (Cross et al., 2021a). Appreciating fully the complexity and multiscale poroelastic features of the brain parenchyma, and related implications relating to skull loading (Gou et al., 2020). The calvarial bones Poisson’s ratio was 0.22, while the sutures were assumed to be 0.3. Both the craniotomy and ICV had a Poisson’s ratio of 0.1. Techniques adopting bioabsorbable fixators (for a particular technique, i.e., TCR1—see simulation section) had an initial elastic modulus and Poisson’s ratio of 2000 MPa and 0.1, respectively (Landes et al., 2006). A sensitivity study analysing the impact of incorporating fixators on morphological shape predictions is presented in the Supplementary Figure S1. To minimise rigid displacement, nodal constraints in all degrees of freedom across the nasal ridge and around the foramen magnum were maintained throughout all simulations. To simulate the rapidly growing brain, we expanded the ICV from the initial 4 months (659 ml) up to 76 months of age (1,376 ml) using a linear thermal expansion analogy across six load steps. At each load step, the age was estimated by correlating the predicted volume against comparative age to volume literature data (Likus et al., 2014).

Establishing the brain growth across the model generated various levels of strain across the sutures and craniotomies, which were used to simulate the bone formation and differentiation of bone stiffness. Here, a two criteria system was parameterised: 1) Applicable elements must achieve a predetermined level of hydrostatic strain (i.e., summation of all principal strains and division by three) as a result of the growth; 2) Elements were required to be within a specified radial distance from the adjoining bone borders. Elements exclusively relative to the sutures were confined to both of these conditions, whilst elements representing the craniotomy followed only the former criteria. To represent the differentiation of bone stiffness, these elements would have their elastic moduli updated, which varied based on the relative changes in age. See our previous studies for a full detailed description of this approach (Cross et al., 2021a,b).

To account for the contact conditions across the ICV, a Hertzian contact algorithm was implemented between the inner-calvarial table and ICV interfaces. The penalty-based behaviour with a normal contact stiffness of 50 N/mm, a penetration tolerance of 0.5 mm and a friction coefficient of 0.1 allowed for minimal levels of interpenetration between surfaces (Malde et al., 2020; Cross et al., 2021a,b). While initially in contact, normal/tangential separation was granted during simulated growth. Interfaces between the calvarial bones and sutures, calvarial bones and craniotomies, and suture and craniotomies maintained a “bonded” contact, restricting all forms of separation.

### Simulated Surgical Techniques

Nine techniques were replicated across the model at various ages of intervention (see also: Gailey et al., 2021). To represent an intervention age greater than the initial 4 months, growth was modelled across three load steps, with no correction having been performed, resulting in three additional models at 6, 9 and 12 months of age. The changes in morphology and contact pressure during the growth are highlighted in the Supplemental Figure S2. The techniques denoted as Renier’s “H,” modified Renier’s “H,” endoscopic, strip cranioplasty, and two variations of spring-assisted craniectomy (SAC) which consisted of two or three springs, respectively (i.e., 2 SAC & 3 SAC) were replicated across the 4 months of age model. For comparison, the Renier’s “H” was also performed across the alternative 6 months of age model. The model at 12 months of age was further used for both total calvarial remodelling techniques (i.e., TCR 1 & TCR 2).

## Results

**FIGURE 2:**
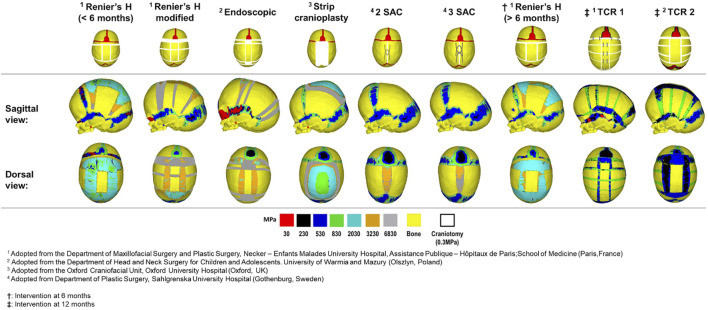
Predicted bone formation across all replicated techniques at the postoperative age of 76 months. The material properties across the newly and previously selected elements were updated at each load step.

First, we qualitatively assessed the pattern of bone formation across all techniques at 76 months of age (Figure 2). Here, the timing of calvarial bone healing was defined as when no respective craniotomy (i.e., white) or suture (i.e., red) elements remained visible across the models. We found that rapid calvarial healing/bone formation at the craniotomies could be achieved by 5 months postoperatively for the SAC techniques, perhaps due to the shorter bilateral width. Followed by the endoscopic treatment at 9 months after surgery. The remaining techniques performed at 4 months of age achieved calvarial healing by 20 months after surgery. When postponing the timing of intervention (i.e., Renier’s “H” at 6 months), little difference in the level and rate of calvarial healing was observed vs. earlier intervention. However, evidence of delayed healing was obtained for both TCR methods; having healed by 24–36 months after surgery. Complete fusion of all sutures was predicted in the modified Renier’s “H,” strip cranioplasty, and both SAC approaches by 76 months of age. Conversely, patency was still visible across the coronal and squamosal sutures in the Renier’s “H” and endoscopic methods at 76 months, respectively. Interestingly, this characteristic was not evident in the later performed Renier’s “H.” Delayed bone formation was predicted across the calvaria in TCR 2 as opposed to TCR 1 (perhaps attributed to the modelled bioabsorbable fixators in TCR 1).

**FIGURE 3:**
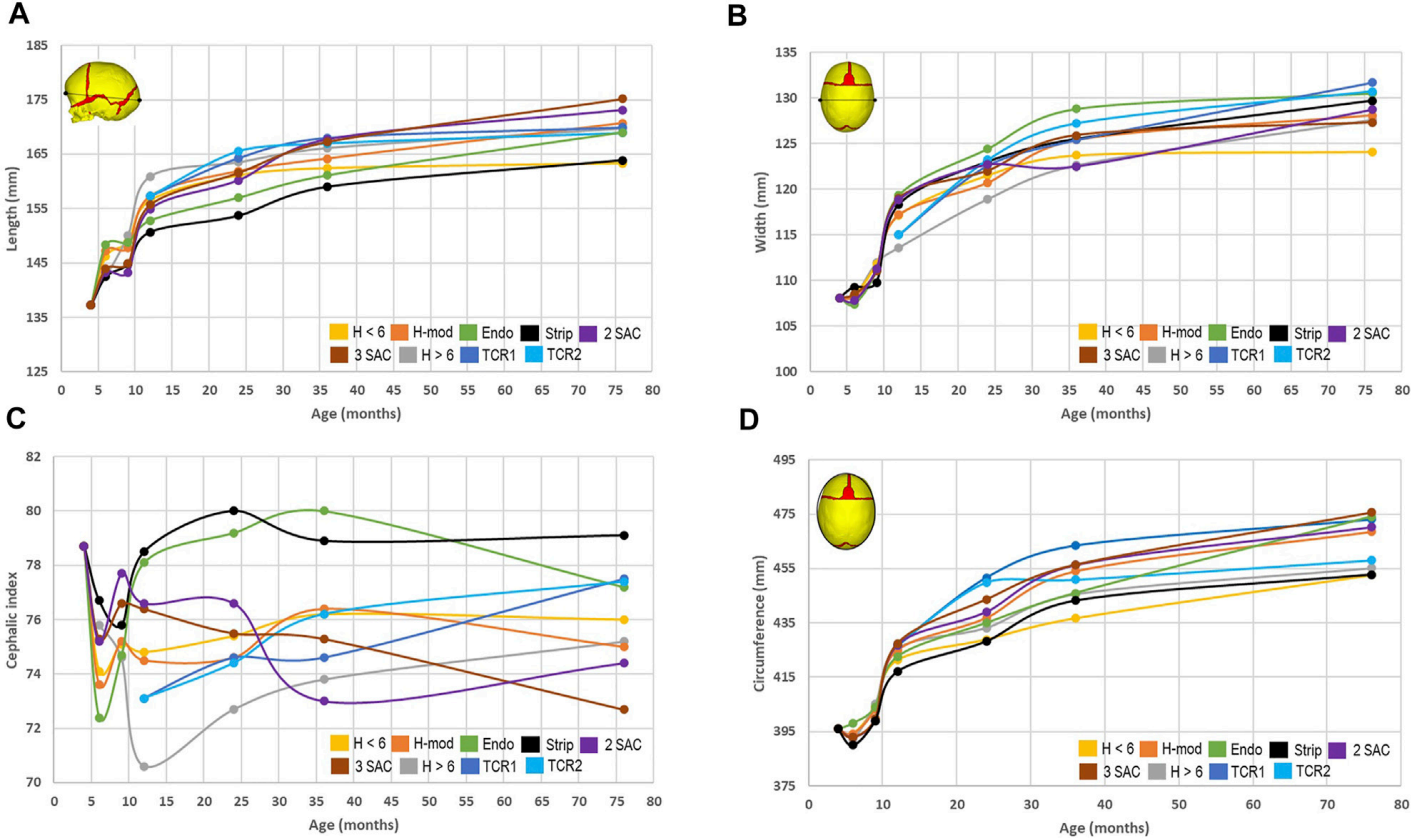
Cephalometric measurements across all replicated approaches. The predicted length **(A)** and width **(B)** were used to calculate the cephalic index **(C)**. The circumference **(D)** was measured in the transverse plane as shown within the diagram.

To quantify the shape changes, we recorded cephalometric parameters for all approaches (Figure 3). By 76 months of age, we predicted the largest overall length in both SAC techniques (Figure 3A), ranging from 175.2 to 173.1 mm, whilst the earlier performed Renier’s H measured 163.3 mm, demonstrating the overall lowest length. Conversely, the greatest bitemporal widening (Figure 3B), measuring 131.7 mm, was achieved in the TCR 1 approach and the least, measuring 124.1 mm, was seen in the earlier Renier’s H. Utilising these values, we calculated the cephalic index by multiplying the width against the length and dividing by one hundred (Figure 3C). Here, with a value of 79.1, the strip craniectomy predicted the overall best improvement with the caveat of a high vertex (see: Figure 2). The worst cephalic index was seen in the 3 SAC predictions, valued at 72.7. We predicted that both total calvarial remodelling techniques, while achieving the second and third best cephalic index values, also showed a reduced level of pre to postoperative relapse in contrast to all earlier techniques. Further, the same response was also seen in the later performed Renier’s H approach. It should be noted however that no technique was able to bring the cephalic index fully back to normal, seen within the normocephalic population at a value of greater than 80 (Gailey et al., 2021; Sgouros et al., 1999). The lowest circumference (Figure 3D) was achieved in the earlier Renier’s H, whilst the greatest was seen in the 3 SAC.

**FIGURE 4:**
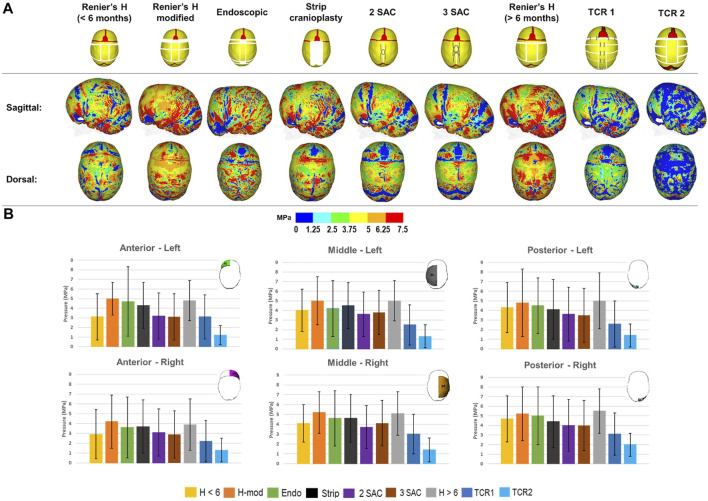
The predicted contact pressure captured across the brain surface for all replicated techniques at 76 months **(A)**. Contact pressure was quantified across different regions of the ICV for each replicated technique with standard deviations corresponding to the nodal distribution across the highlighted regions **(B)**. All results were recorded at 76 months of age.

Using pressure maps, we qualified the predicted contact pressure across the ICV at 76 months for all techniques (Figure 4A). To quantify these predictions, we subdivided the ICV component for all techniques into six regions of interest, with standard deviations highlighting the differences in pressure in these regions across the entire area (Figure 4B). The anterior, middle and posterior cerebral areas were divided between the left and right sides. We predicted that the TCR 2 approach leads to the lowest consistent pressure outcome across the anterior (Left: 1.2 ± 1.0 MPa–Right: 1.3 ± 1.5 MPa), middle (Left: 1.3 ± 1.2 MPa–Right: 1.4 ± 1.2 MPa) and posterior (Left: 1.4 ± 1.2 MPa–Right: 2.0 ± 1.2 MPa) regions of the ICV. In an earlier intervention, the modified Renier’s “H” approach estimated the overall greatest pressure values across the left (5.0 ± 1.7 MPa) and right (4.2 ± 2.7 MPa) anterior regions. Similar pressure findings were seen between the modified Renier’s “H” and the later performed Renier’s “H” procedure, which consistently produced the largest values across the middle left (modified Renier’s: 5.0 ± 2.5 MPa–Renier’s H: 5.0 ± 2.1 MPa), middle right (modified Renier’s: 5.2 ± 2.1 MPa–Renier’s H: 5.1 ± 2.2 MPa), posterior left (modified Renier’s: 4.8 ± 3.5 MPa–Renier’s H: 5.0 ± 2.9 MPa) and posterior right regions (modified Renier’s: 5.2 ± 2.8 MPa–Renier’s H: 5.5 ± 2.3 MPa). Interestingly, the number of simulated springs across both 2 SAC and 3 SAC techniques did not impact the overall pressure predictions, despite the small differences observed in the cephalometric measurements.

## Discussion

Clinically, the target for SS correction is anteroposterior reduction accommodated by mediolateral and dorsal expansion. The computational framework proposed here showed that all techniques achieve this underlying objective yet with different morphological outcomes and relapses. The predicted morphologies and the contact pressure maps obtained across the calvaria highlighted that different reconstructive techniques constrain/facilitate the growth of the brain to a different extent.

Our results overall highlighted that the more invasive techniques (TCR 1 & 2) can potentially lead to a higher cephalic index compared to the less invasive techniques by 76 months of age. Further, the predicted contact pressure maps highlighted a lower level of pressure over the surface of the expanding brain for the two considered total calvarial remodelling techniques as opposed to the other considered techniques. Interestingly, our analysis of clinical CTs of over 100 scans corresponding to the techniques investigated in this study did not find a statistically significant difference in the morphological outcome of these techniques (Gailey et al., 2021). However, it must be re-emphasised that the framework presented here does not take into account any clinical variables potentially differentiating the different cases (Gailey et al., 2021) regarding the calvarial reconstruction techniques. This can be interpreted as both an advantage of the framework presented here and also as its key limitation.

Indeed, assumptions had to be made in the proposed computational framework. Many chemical and biological characteristics play a role in membranous bone formation during infancy (Opperman, 2000; Herring, 2008; Richtsmeier & Flaherty, 2013; Beederman et al., 2014) while a purely mechanical approach was considered here. Even within the considered approach, we have not incorporated a more detailed description of the hydrostatic loads caused by the normal skull and brain growth. Further, normal growth is most probably non-linear and anisotropic, versus the linear isotropic approach adopted here. Nonetheless, since this approach was uniformly applied to all techniques that were modelled, we were able to achieve a similar level of ICV volume size and shape changes up to 76 months of age (Gailey et al., 2021; Sgouros et al., 1999). Hence, while we cannot be confident in the exact *absolute* values reported in this study, the relative comparisons provide invaluable insights for years to come.

Our previous studies have assessed the impacts that alternative material properties could have on the predicted calvarial growth (Cross et al., 2021a). As such, these properties were brought forward to the current study, including the method of uniformly updating the elastic modulus of the bone. Whilst the effects of changing the elastic modulus of the calvarial bones were assessed, the current model lacks the consideration that viscoelastic properties could have on the manipulation of the bone morphology (Margulies and Thibault, 2000). Such impacts have been assessed previously and are an important consideration for computational models when replicating the after-effects of surgery (Borghi et al., 2018; Borghi et al., 2020). However, as we believe this only plays a role across a small time scale (perhaps within hours post-operatively), the former method of replicating the changes in bone properties was chosen (considering that we predicted the skull growth up to 76 months of age).

The assessment of cognitive outcomes pre-and post-operatively across differing techniques is typically conducted using dedicated questionnaires. Within the literature, there is still debate as to the optimum treatment option based on the outcomes of such questionnaires (Hashim et al., 2014; Care et al., 2019; Kljajic et al., 2019). The contact pressure data obtained in our work is a surrogate to estimate to what extent different techniques constrain the growth of the brain parenchyma. The exact values predicted here must be treated with caution and require further investigations and validation, yet, they may prove informative for craniofacial surgeons in a comparative manner and provide a level of postoperative cognitive predictability when considering treatment options.

Clinically, the choice of a treatment option needs to be optimised based on a number of factors such as the experience of the team in performing a specific technique, the necessity of blood transfusion (Meyer et al., 1993) and various associated costs. These factors were not considered in the computational framework proposed here. Further, more work is required to implement facial growth in the proposed approach. Orbital, mid-facial and palate deformations most probably play a role in calvarial morphometric outcomes (Ranly, 2000).

In summary, we believe the presented approach provides a sustainable way of assisting with preoperative sagittal craniosynostosis management and estimating the postoperative outcomes. The potential to examine the changes in biomechanical behaviour allows for the optimisation of morphological and cognitive characteristics in patients’ years after surgery. This, in the long term, can reduce the level of complications and improve the overall quality of care.

## Data Availability

The original contributions presented in the study are included in the article/Supplementary Material, further inquiries can be directed to the corresponding author.
