# Supplementary material for: A Computational Framework to Predict Calvarial Growth: Optimising Management of Sagittal Craniosynostosis
Source: Front Bioeng Biotechnol. 2022 May 24;10:913190. doi: 10.3389/fbioe.2022.913190 (PMC9170984; doi:10.3389/fbioe.2022.913190)
Supplement: Supplementary file 1 [file DataSheet1.docx]

**
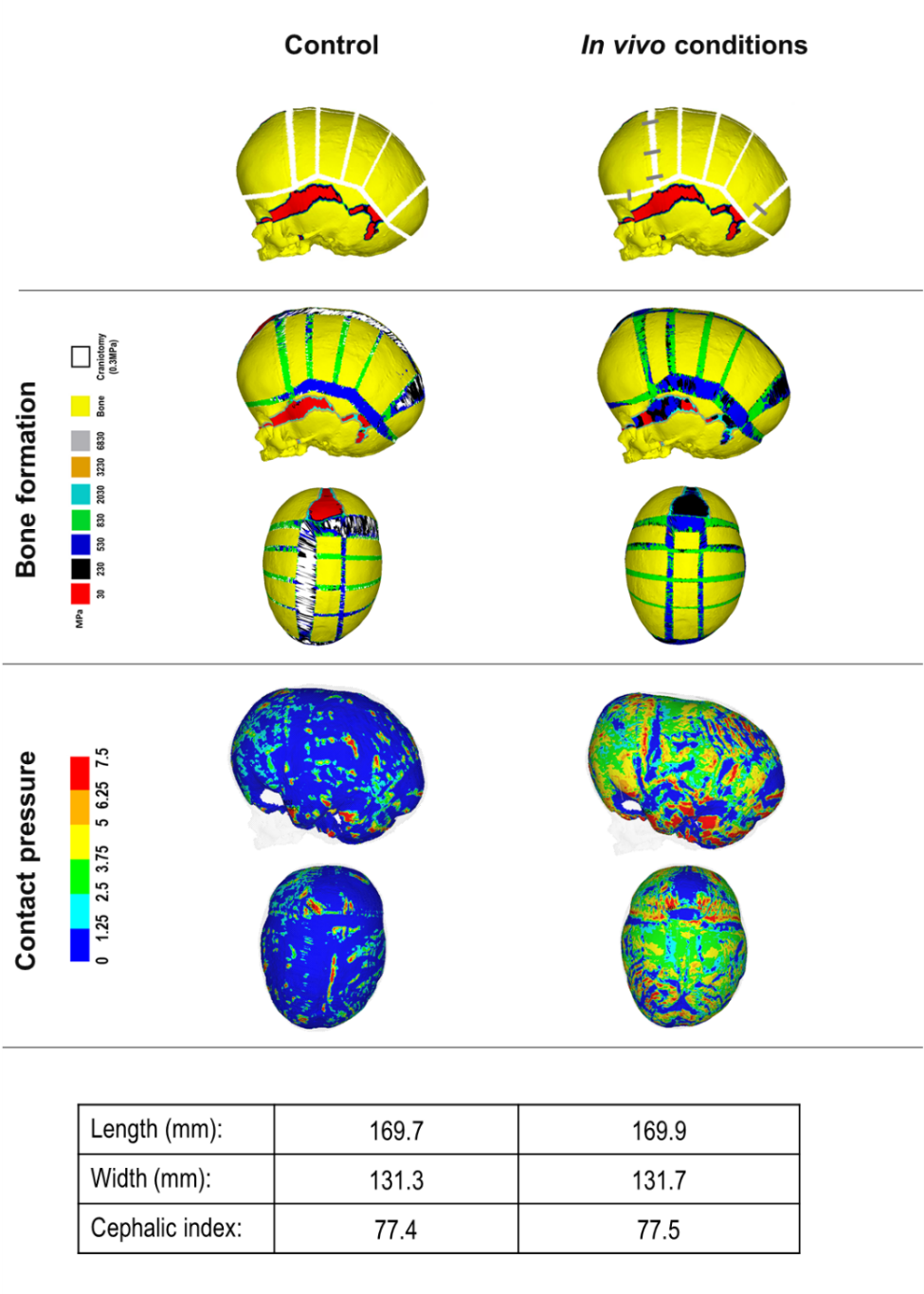
Supplement:**

**Fig. S1:** Sensitivity results on the effects of simulating bioabsorbable fixators across the TCR 1 corrective technique at 12 months of age (Top row). We compared this with a control scenario, in which such fixators were absent. We specified the elastic modulus of the fixators as 2000 MPa. The fixators were then parameterised to be removed by 12 months after insertion, to represent the degradation of the fixators. Here, we captured a large change in the level of bone formation across the two approaches by 76 months of age (Middle row), in which a lack of fixators delayed the rate of formation across the temporal and anteroposterior craniotomies. We further observed a larger increase in the brain contact pressure (Bottom row) when fixators were present. Although large changes between these scenarios were captured, changes in cephalic measurements (See table) displayed little overall change.


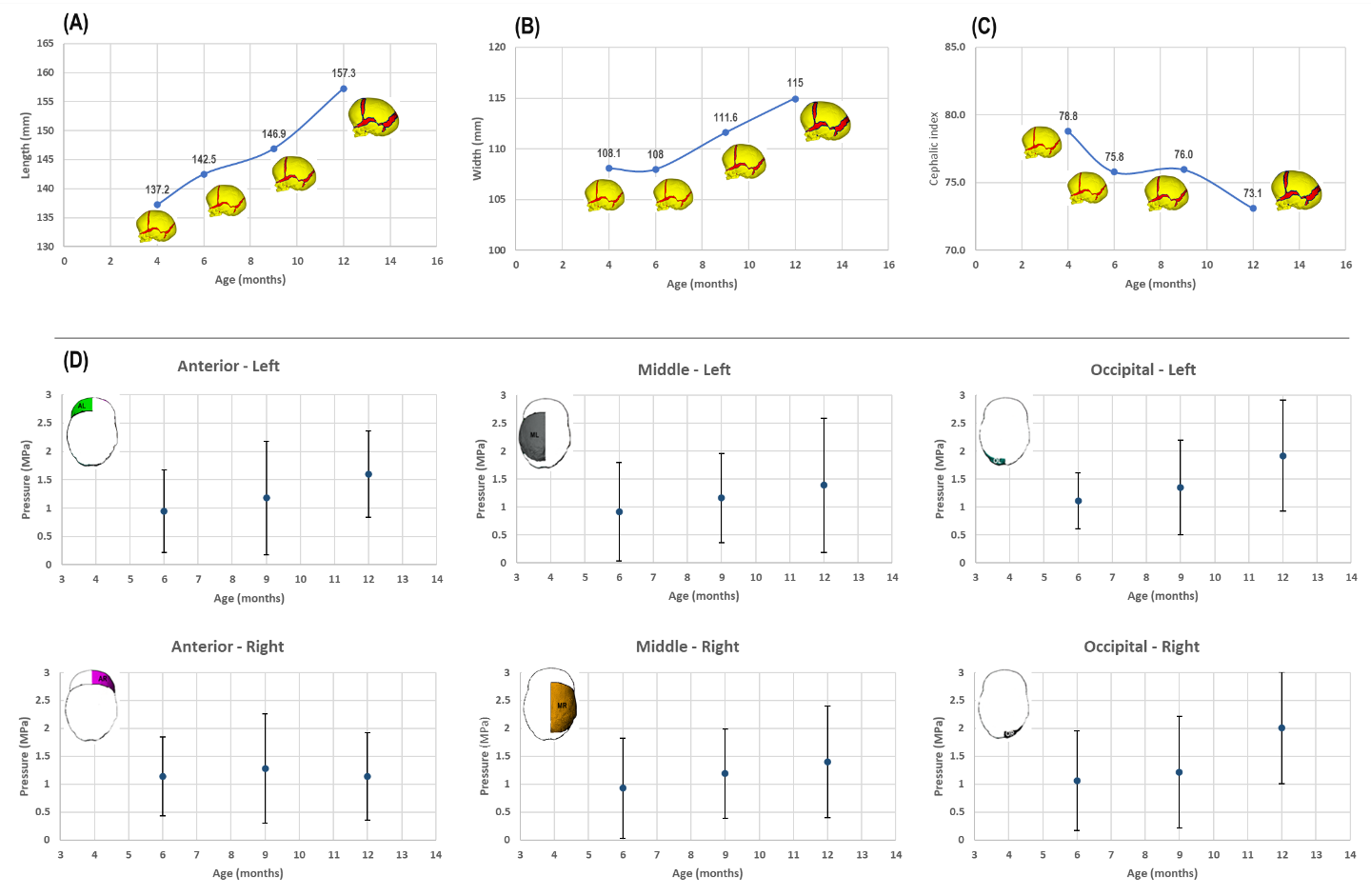


**Fig. S2:** Cephalometric measurements (Top row) under an unoperated condition up to 12 months of age, enabling us to replicate techniques later than the initial 4-month geometry. A greater length (A) was captured whilst a reduced width (B) was achieved, leading to a lower cephalic index with increasing age (C). Interestingly, the level of contact pressure was seen to be asymmetrical across the anterior regions, whilst the largest pressure levels were captured across the occipital area (D).
